# Hierarchical Prototype Alignment for Video Temporal Grounding

**DOI:** 10.3390/e28040389

**Published:** 2026-04-01

**Authors:** Yun Tian, Xiaobo Guo, Jinsong Wang, Yuming Zhao, Bin Li

**Affiliations:** 1 School of Optoelectronic Engineering, Changchun University of Science and Technology, Changchun 130022, China; 2 School of Mechanical and Electrical Engineering, Suqian University, Suqian 223800, China; 3 Jiangsu Engineering Research Center of Key Technology for Intelligent Manufacturing Equipment, Suqian 223800, China; 4 Shenzhen Institute of Advanced Technology, Chinese Academy of Sciences, Shenzhen 518055, China

**Keywords:** video temporal grounding, prototype learning, cross-modal alignment

## Abstract

Recent advances in vision-language cross-modal learning have substantially improved the performance of video temporal grounding. However, most existing methods directly associate global video features with sentence-level features, overlooking the fact that textual semantics usually correspond to only limited spatio-temporal regions within a video. This limitation often leads to unstable alignment in complex scenarios involving intertwined events and diverse actions. In essence, accurate video temporal grounding requires the joint modeling of fine-grained spatial semantics and heterogeneous temporal event structures. Motivated by this observation, we propose a hierarchical prototype alignment approach that models cross-modal correspondence between video and text through structured intermediate prototype representations. Specifically, the alignment process is decomposed into two complementary stages: object-phrase alignment and event-sentence alignment. In the object-phrase alignment stage, discriminative local visual regions and informative textual words are aggregated to construct object and phrase prototypes, thereby enhancing fine-grained spatial correspondence at the level of entities and localized actions. In the event-sentence alignment stage, object prototypes are further integrated along the temporal dimension to form event prototypes that represent continuous action units, enabling effective alignment with sentence-level semantics and facilitating the modeling of diverse temporal event structures. On this basis, we further directly inject cross-modal alignment information into candidate moment aggregation. This design allows candidate moment representations to emphasize query-relevant temporal regions. Extensive experiments on Charades-STA, ActivityNet Captions, and TACoS demonstrate that the proposed method outperforms existing approaches, validating the effectiveness of hierarchical prototype alignment for improving both cross-modal alignment quality and temporal grounding accuracy.

## 1. Introduction

Understanding multimodal information [1,2,3] is a fundamental capability that enables computers to perceive and interpret the world. As a core task in multimodal learning [4,5,6], video temporal grounding (VTG) has broad applications in multimodal retrieval, intelligent surveillance, and human–computer interaction, and has therefore attracted significant research attention. VTG [7,8] is defined as the task of localizing a temporal segment within an untrimmed video that corresponds to a given natural language query. Situated at the intersection of computer vision (CV) and natural language processing (NLP), VTG serves as a fundamental component in various video understanding tasks, including video dialogue [9], relationship detection [10,11,12], and question answering [13,14,15].

**Figure 1:**
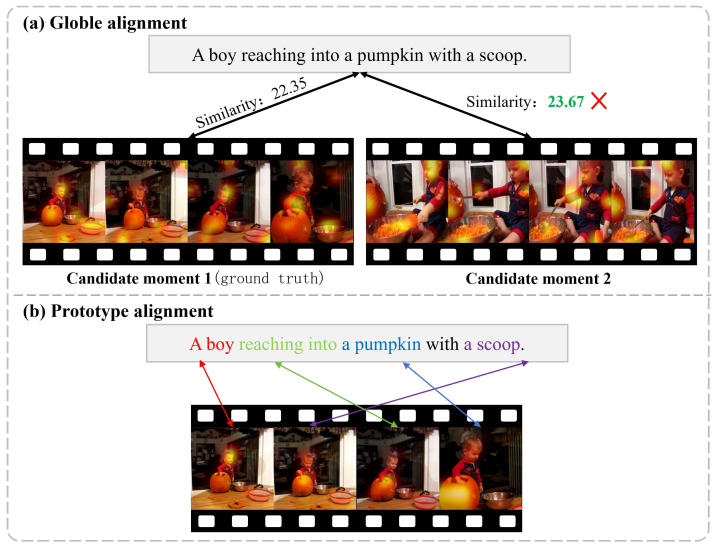
Comparison between global alignment and prototype alignment for video temporal grounding. (**a**) Global alignment assigns similarity scores based on holistic video–sentence representations, which may incorrectly favor visually similar but semantically mismatched candidate moments. The red multiplication sign (×) indicates an incorrect candidate moment, arrows denote similarity matching relationships, and different colors represent different similarity values. (**b**) Prototype alignment decomposes the sentence into object- and action-related semantic units and aligns them with localized visual regions, enabling fine-grained correspondence between textual phrases and video content and leading to more reliable moment discrimination.

Videos contain complex spatio-temporal dynamics, involving evolving scenes, entities, and events. In contrast, natural language is inherently abstract and ambiguous, where a single sentence may refer to different combinations of actions and objects. This inherent modality gap [16,17,18] increases the difficulty of associating multimodal data and complicates feature integration in video temporal grounding. In recent years, many studies have focused on improving video-text alignment for temporal localization tasks. Several existing methods [19,20,21,22,23] represent both video and query as single embeddings and perform matching within a global semantic space. While these approaches attempt to establish a holistic semantic correspondence between the video and the query, in practical scenarios, the query usually refers to a specific spatio-temporal segment, which makes global matching susceptible to interference from irrelevant content. To improve matching granularity, later works [24,25,26,27,28] introduce frame-level or segment-level matching strategies that assign different weights to frames or segments for fine-grained alignment, thereby improving discriminative capability. However, videos contain rich visual elements, and a text description may correspond only to a spatio-temporal portion of the video. Consequently, dynamic and fine-grained matching is required beyond global video-level or coarse frame-level matching. More specifically, feature encoding should represent semantically diverse components to support localized and context-sensitive alignment during the matching process. To address this issue, several studies attempt to incorporate intermediate representations to mitigate semantic inconsistencies. For example, Lin et al. [29] decompose videos into semantic events and introduce event prototypes that enable flexible alignment between queries and temporal event structures. As illustrated in Figure 1a, the query specifies not only the action but also the tool and object, and its semantics do not correspond to the entire scene but depend on several localized visual cues. If alignment relies only on global features or coarse event representations, the model may overlook local evidence or become sensitive to background distractions, which may lead to misalignment [30,31].

**Figure 2:**
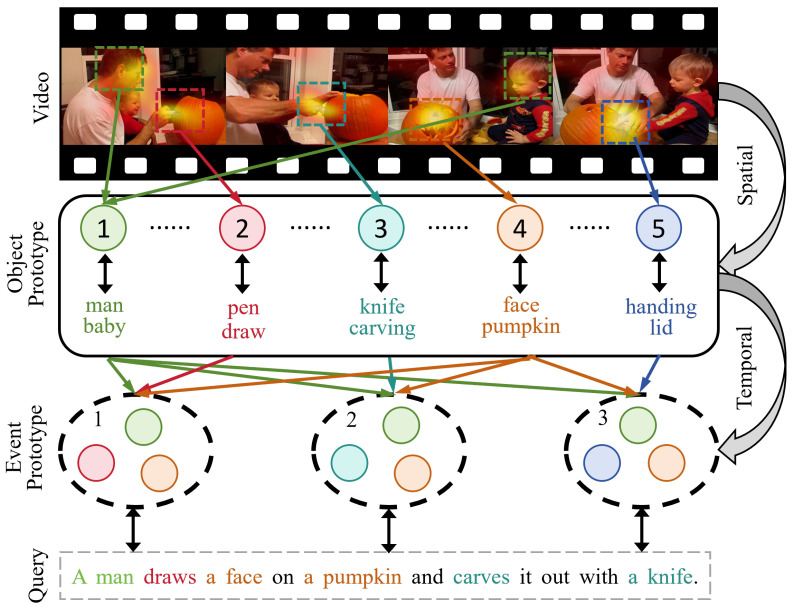
Illustration of the hierarchical prototype alignment process. Object prototypes are first constructed by attending to spatial regions within frames, capturing fine-grained entity cues. These object cues are then progressively aggregated along the temporal dimension to form event prototypes, which model action evolution and support sentence-level semantic alignment. Different colors represent different prototype groups, numbers denote prototype indices, arrows indicate the information flow between components, and ellipses represent intermediate prototypes.

Based on these observations, we argue that reliable video-text alignment should be grounded in fine-grained local semantics and supported by a structurally stable intermediate representation. Such a representation carries multi-level semantics, preserves necessary spatial correspondence, and progressively integrates temporal context to facilitate hierarchical cross-modal alignment. However, local entity cues and temporal event structures are inherently heterogeneous. Spatial semantics originate from transient visual regions, whereas event-level meanings emerge from temporal aggregation across frames. Modeling them within a single unified representation may blur this distinction, allowing dominant temporal patterns to overshadow localized evidence or causing spatial noise to disrupt temporal coherence. To address this structural mismatch, we organize the alignment process along both spatial and temporal dimensions through a spatio-temporal prototype formulation. We first generate object prototypes in the spatial dimension by aggregating salient local regions to capture phrase-related entities and local action cues. As shown in Figure 1b, object and phrase prototypes enable the model to identify key local regions while suppressing irrelevant background content. On this basis, we further aggregate object prototypes into event prototypes so that the resulting prototypes represent not only within-frame semantics but also the temporal continuity of basic action units. This two-stage prototype representation forms a coherent structure that bridges local and temporal semantics, thereby providing a more stable and explicit foundation for cross-modal alignment. Figure 2 shows that our method progressively constructs cross-modal prototypes across multiple semantic levels, which improves the model’s ability to capture complex semantic structures. Specifically, the spatial prototype module detects semantically salient local regions to form object prototypes, such as a man and a baby. This process reduces visual noise and strengthens fine-grained alignment between visual features and textual phrases. Furthermore, object prototypes are aggregated into event prototypes to represent cross-frame semantics, such as drawing on a pumpkin and carving with a knife, which correspond to complete action sequences or sentence-level semantic units.

In addition, existing approaches typically construct candidate moment representations by pooling or convolutional aggregation of frame features across the candidate interval, a design that often struggles to emphasize key semantic regions in the presence of background redundancy or loosely related motions. By contrast, we treat the spatio-temporal prototype matching scores as semantic weights and perform weighted fusion across temporal positions within each candidate interval, thereby aligning the representation construction process with the cross-modal alignment results. The resulting moment representations place greater emphasis on temporal regions relevant to the query, providing more informative inputs for boundary prediction and alleviating the lack of semantic selection in conventional moment representation learning.

The main contributions of this work are summarized as follows:

- We propose a hierarchical prototype alignment method that decomposes cross-modal alignment into two complementary stages, namely object-phrase alignment and event-sentence alignment, thereby improving alignment accuracy from both spatial and temporal perspectives.
- We develop two prototype aggregation networks to model spatial and temporal information in videos, enabling the framework to capture fine-grained local details and diverse semantic patterns.
- Instead of constructing moment candidates through pooling or stacked convolution, we introduce an alignment result to construction strategy that emphasizes temporally localized regions most relevant to the textual semantics.
- Experiments on Charades-STA, ActivityNet Captions, and TACoS demonstrate that the proposed method outperforms existing approaches across multiple evaluation metrics.

## 2. Related Work

### 2.1. Video Temporal Grounding

A central challenge in video temporal grounding lies in cross-modal alignment, which requires mapping the video stream to a natural language query in a semantically consistent manner over time. A common line of work formulates localization as global semantic matching by embedding the entire video and the complete sentence into a shared space and using video-sentence similarity to select the target segment. Methods including VSLNet [21], 2D-TAN [19], GDP [32], CPN [23], and DCM [33] generally rely on global video representations or candidate semantic fusion. These approaches typically encode the video as a whole and then score moment candidates in a uniform manner. Such strategies can be effective when the overall semantics are highly consistent. However, natural language queries often describe only a local event or a key action. As a result, global alignment inevitably introduces query-irrelevant visual content and may lead to semantic drift in long videos or complex scenes.

To mitigate the interference introduced by global modeling, some studies incorporate finer temporal modeling or weighting mechanisms within global frameworks. For example, multi-scale designs, cross-modal attention, and candidate enhancement strategies have been adopted to strengthen responses in target regions. MMN [20], PS-VTG [34], ViGA [35], D-TSG [36], and D3G [37] extend this direction by strengthening video-text interaction or introducing structured reasoning mechanisms, which improves localization accuracy to some extent. However, in these approaches cross-modal alignment is often absorbed into fusion or prediction modules rather than being explicitly modeled in terms of the visual structures referred to by the text. Consequently, their ability to represent complex semantic composition remains limited.

With continued progress, recent research increasingly emphasizes fine-grained cross-modal alignment by linking local visual content to discrete semantic units in language. Methods such as LGI [25], MAT [38], and DRN [39] introduce interaction mechanisms between frame-level or region-level cues and textual phrases, enabling the model to capture the visual evidence referred to by the query more precisely. Later works including LCNet [40], CBLN [24], and SeqPAN [27] further strengthen the temporal consistency of local semantic alignment by improving action boundary modeling through hierarchical designs or sequence-aware mechanisms. Recent studies such as MGSL [28], PFU [41], VDI [42], and MESM [26] refine local alignment through frame-level supervision, modality balancing, or semantic enrichment, which improves robustness under challenging conditions. Meanwhile, recent studies have begun exploring large language model (LLM)-based paradigms for multimodal temporal understanding and reasoning, such as PromptCC [43] and Change-Agent [44], which leverage pretrained language models to facilitate semantic interpretation and reasoning over temporal imagery.

Although local alignment approaches substantially improve sensitivity to fine-grained semantics, most existing studies focus on alignment at a single level, such as frame-to-word or region-to-phrase correspondences, and lack a systematic characterization of video semantics spanning from local entities to temporal events. When a query jointly involves objects, actions, and their temporal relations, relying on a single level of local alignment often fails to provide stable and coherent cross-modal support. A key question in video temporal grounding is how to retain local precision while introducing an intermediate representation that captures semantic hierarchy and enables complementary alignment across multiple levels. To address this issue, we propose hierarchical prototype alignment to strengthen cross-modal consistency between video and text.

### 2.2. Prototype Learning

Prototype learning traces its origins to early developments in machine learning and cognitive psychology, where the central idea is to represent each class with a prototypical exemplar and perform inference based on distance metrics between the input and these prototypes [45,46,47]. In the domains of few-shot and zero-shot learning, prototype-based approaches have attracted significant attention due to their strong generalization capability [48,49]. For example, Gao et al. [50] introduced a hybrid-attention prototype network for relation classification with noisy labels, and Zhao et al. [51] proposed a knowledge-augmented prototype architecture to improve few-shot event detection. These studies demonstrate that it improves the interpretability and consistency of semantic alignment.

More recently, prototype learning has been increasingly adopted in multimodal contexts, showing strong capability in modeling structured semantics and facilitating cross-modal alignment. In the context of event relation extraction, Hu et al. [52] proposed the Prototype-Enhanced Matching (ProtoEM) framework, which models prototype nodes for event types and encodes their semantic dependencies through graph neural networks, thereby improving the recognition of structured event relations. Huang et al. [53] addressed few-shot action recognition by introducing a compound prototype matching method, where videos are represented as a set of compound prototypes, including both global and local prototypes. Their method integrates one-to-one and bipartite matching to enhance temporal alignment at multiple scales. For video-text retrieval, Lin et al. [29] proposed a Text-Adaptive Multiple Visual Prototype Matching framework, in which multiple visual prototypes are learned to represent diverse video semantics. The model performs text-adaptive matching by selecting the most relevant prototype for each query, and a variance loss is introduced to encourage prototype diversity.

Although these studies demonstrate the effectiveness of prototype-based modeling in multimodal tasks, its application to video temporal grounding remains relatively limited, particularly in integrating hierarchical semantic structures with temporal localization. Moreover, in most existing approaches, prototypes are mainly used to improve representation learning or similarity matching, serving as intermediate semantic abstractions that facilitate feature alignment across modalities. In contrast, the proposed framework incorporates hierarchical prototype alignment directly into the temporal grounding process. In this work, we extend prototype modeling to video temporal grounding by proposing a hierarchical prototype alignment method.

## 3. Methodology

**Figure 3:**
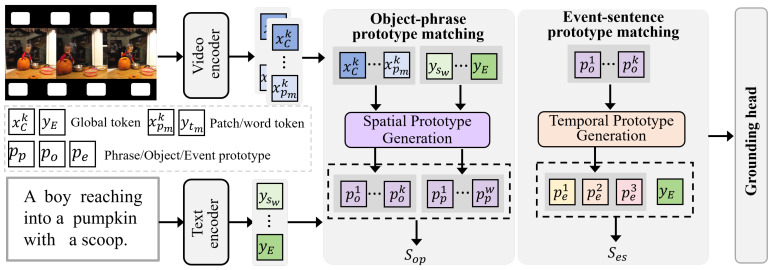
Overview of the proposed hierarchical prototype alignment framework. We decompose cross-modal alignment into two complementary components: (1) Object-Phrase Prototype Matching (OPPM), which aligns visual object prototypes and textual phrase prototypes generated by Spatial Prototype Generation (SPG) to emphasize fine-grained spatial semantics; (2) Event-Sentence Prototype Matching (ESPM), which exploits event prototypes progressively generated by Temporal Prototype Generation (TPG) to model temporal action evolution and establish coherent alignment with sentence-level semantics. The resulting matching scores are further integrated by the grounding head for temporal boundary prediction. Overlapping elements represent multiple video frames in the sequence.

This work aims to strengthen cross-modal alignment between video and text, and the overall framework is illustrated in Figure 3. The framework consists of a multimodal backbone and a two-stage prototype alignment module. The backbone comprises a video encoder and a text encoder that extract visual and textual representations in a shared semantic space. Building on these representations, we further generate object and event prototypes as intermediate representations across semantic levels, allowing alignment to progress from local entities to temporal event units.

### 3.1. Problem Definition

Given an untrimmed video *V* and a natural language query sentence *S*, video temporal grounding (VTG) aims to localize a temporal interval *E* in *V* whose visual content is most semantically consistent with *S*. The query sentence *S* is represented as a sequence of words $S={\left\{s_{i}\right\}}_{i=0}^{W-1}$, where $s_{i}$ denotes the *i*-th word and *W* is the total number of words in the sentence. The video *V* is represented as a sequence of frames $V={\left\{x_{j}\right\}}_{j=0}^{L-1}$, where $x_{j}$ denotes the *j*-th frame and *L* denotes the total number of frames. The target moment is denoted as $E=[t_{s},t_{e}]$, where $t_{s}$ and $t_{e}$ represent the start and end timestamps, respectively. Predicting *E* can therefore be viewed as a cross-modal alignment problem in which the model identifies video evidence that corresponds to the semantics expressed in *S*.

### 3.2. Preparation

In this work, a unified and stable semantic space provides the foundation for prototype formation and cross-modal alignment. Accordingly, we first specify how video and text are represented, and then describe the clip segmentation scheme and the feature encoding process of the backbone. We divide the untrimmed video *V* into *N* fixed-length clips ${\left\{v_{i}\right\}}_{i=0}^{N-1}$, denoted by $V={\left\{v_{i}\right\}}_{i=0}^{N-1}$. Each clip $v_{i}$ consists of *T* consecutive frames, expressed as $v_{i}={\left\{x_{j}\right\}}_{j=t_{i}}^{t_{i}+T-1}$, where $x_{j}$ is the *j*-th frame and $t_{i}$ is the starting frame index of the *i*-th clip. Since key actions may occur near the boundary between adjacent clips, we adopt a frame-overlap strategy to avoid truncating critical information, keeping *O* overlapping frames such that $t_{i+1}=t_{i}+\left(T-O\right)$. With this segmentation, the number of clips *N* is computed as

(1) $$N=\left\lfloor \frac{L-O}{T-O}\right\rfloor +1.$$

If the tail segment contains fewer than *T* frames, we use zero padding to enforce a fixed clip length, which keeps the feature dimensions consistent for later encoding and prototype alignment. The *N* sampled clips serve as the basic units for constructing moment candidates.

In the feature-encoding stage, we adopt CLIP [54] as the multimodal backbone, which establishes a shared semantic space for vision and text via cross-modal contrastive learning. For each video segment $v_{i}$, we uniformly sample *K* frames and feed them into the CLIP visual encoder to obtain the visual feature sequence:

(2) $$X_{k}={\left\{x_{c}^{k},x_{p_{1}}^{k},\ldots ,x_{p_{M}}^{k}\right\}}_{k=1}^{K}\in R^{K\times (M+1)\times D}.$$

Here, $x_{c}^{k}$ denotes the global token of the *k*-th frame and summarizes its overall semantics. The set ${\left\{x_{p_{m}}^{k}\right\}}_{m=1}^{M}$ represents the local features of the frame, consisting of *M* patch tokens that preserve fine-grained visual cues such as object shape and local motion. After encoding $v_{i}$, the resulting visual representation captures both global semantics and local structure, serving as the visual foundation for cross-modal alignment. For the query text $S={\left\{s_{i}\right\}}_{i=0}^{W-1}$, we extract semantic features using the CLIP text encoder. We prepend [SOT] and append [EOT] to mark the sequence boundaries, and represent the encoded feature sequence as:

(3) $$Y=\left\{y_{S},y_{s_{1}},\ldots ,y_{s_{W}},y_{E}\right\}\in R^{(W+2)\times D},$$

where $y_{E}$ is the global text token feature [EOT], and *W* and *D* denote the number of words and the feature dimension, respectively. Beyond the backbone, we decompose the alignment process into two complementary spatio-temporal components. Object-phrase prototype alignment aligns object prototypes from the spatial prototype generator with phrase prototypes, focusing the model on fine-grained spatial evidence. Event-sentence prototype alignment leverages event prototypes progressively generated by the temporal prototype generator to learn dynamic semantic alignment and capture the intrinsic one-to-many relation between video and text. The matching scores from the object-phrase and event-sentence pathways reflect the overall quality of cross-modal alignment.

### 3.3. Object-Phrase Prototype Alignment

In video temporal grounding, the query text typically describes events within a video segment, and semantic elements such as objects and actions often correspond to local regions in the video. These local regions contain key visual cues for cross-modal alignment, but they are also mixed with substantial background distractions and irrelevant details. Relying only on global features or simple frame-level alignment makes it difficult to establish stable cross-modal correspondences. Therefore, we construct object prototypes and phrase prototypes as spatial prototypes to capture semantic consistency among visual object instances, action-related regions, and textual phrase representations. This section consists of two parts. We first construct video object prototypes and text phrase prototypes, and then evaluate cross-modal alignment based on their semantic similarity.

#### 3.3.1. Spatial Prototype Generation

**Figure 4:**
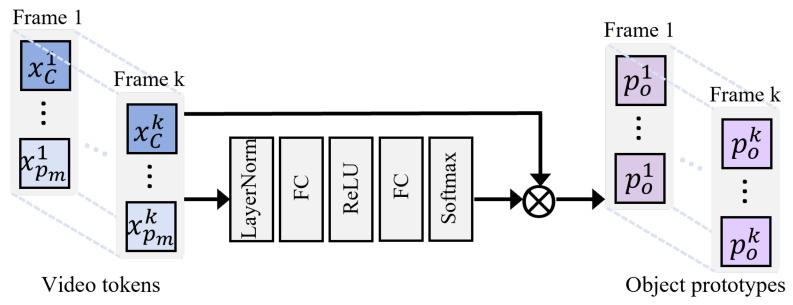
Framework of the Spatial Prototype Generation (SPG) module. Given patch tokens from different frames, SPG learns frame-adaptive attention weights through a lightweight feed-forward network to aggregate informative patches into object prototypes. The generated prototypes emphasize query-relevant spatial regions while suppressing background noise, providing compact and discriminative representations for subsequent object-phrase prototype matching.

After the video segment is encoded by the visual encoder in the previous subsection, the resulting visual feature sequence contains a global token and local patch tokens. We aggregate patch tokens into object prototypes that represent fine-grained spatial content, such as object instances, parts, and action-relevant regions. Not every patch should contribute to a prototype, since background patches may carry spatial signals but often introduce noise for cross-modal alignment. Therefore, we adopt sparse aggregation to filter redundant information and generate spatial prototypes. The architecture of the Spatial Prototype Generator (SPG) is shown in Figure 4. In the spatial prototype generator, to alleviate training instability caused by feature scale differences across frames, we first apply layer normalization to the *k*-th frame feature sequence $X_{k}\in R^{(M+1)\times D}$:

(4) $${\hat{X}}_{k}=\mathrm{LayerNorm}\;\left(X_{k}\right).$$

Then, based on the normalized features ${\hat{X}}_{k}$, we use a two-layer fully connected network with ReLU to predict the aggregation weight matrix $W_{o}^{k}$ for mapping spatial positions to object prototypes:

(5) $$W_{o}^{k}=W_{2}\;\left(\mathrm{ReLU}\;\left(W_{1}{\hat{X}}_{k}+b_{1}\right)\right)+b_{2}\in R^{\left(M+1\right)\times N_{o}}.$$

Here, $N_{o}$ denotes the preset number of object prototypes. To encourage each prototype to concentrate on a few highly relevant patches and to keep weights comparable across frames, we normalize the scores with Softmax along the spatial dimension, producing the sparse aggregation matrix $A_{k}$:

(6) $$A_{k}=\mathrm{Softmax}\;\left(W_{o}^{k}\right)\in R^{\left(M+1\right)\times N_{o}}.$$

In Equation (6), Softmax normalizes each column *j* along the spatial index *i*, ensuring that $\sum_{i=1}^{M+1}{\left[A_{k}\right]}_{ij}=1.$ The *j*-th column can be viewed as a probability distribution over spatial positions (patches) for the *j*-th object prototype, indicating which patches contribute most to the aggregation. This design encourages each prototype to be supported by a few high-weight patches, thereby suppressing redundant content. Finally, object prototypes are obtained by a weighted aggregation of visual features:

(7) $$P_{o}^{k}={\left(A_{k}\right)}^{⊤}\cdot X_{k}\in R^{N_{o}\times D}.$$

As a result, each object prototype is a *D*-dimensional vector that represents a spatial semantic unit aggregated from several key patches in the *k*-th frame. Ideally, each object prototype can adaptively aggregate patches associated with the corresponding object or action.

For phrase prototypes, since a query contains multiple semantic components, different phrases often correspond to different visual objects or action regions. Therefore, it is necessary to extract referential semantic units from the word sequence rather than aligning visual features with the entire sentence. To this end, we adopt a sparse aggregation strategy analogous to that used on the visual side and design a similar network to aggregate the text feature sequence, producing phrase prototypes $P_{p}\in R^{N_{p}\times D}$, where $N_{p}$ denotes the number of phrase prototypes. Phrase prototypes transform a natural language sequence into a set of localized semantic units. Both object and phrase prototypes are dynamically instantiated from the current visual and textual features through sparse aggregation, rather than maintained as static prototype parameters. During training, the prototype representations are recomputed in each forward pass according to the updated aggregation weights, allowing the prototypes to adapt to the semantic content of each input sample.

#### 3.3.2. Object-Phrase Matching

Object-phrase alignment seeks object prototypes in a clip whose semantics most strongly coincide with a phrase prototype across frames. To measure such correspondence, for each frame *k* we construct an object-phrase prototype similarity matrix using dot-product similarity:

(8) $$M^{k}=P_{p}\cdot {\left(P_{o}^{k}\right)}^{⊤}\in R^{N_{p}\times N_{o}}.$$

Each element ${\left[M^{k}\right]}_{ij}$ measures the similarity between phrase prototype *i* and object prototype *j* at the *k*-th frame. For a fixed object index *j* and frame index *k*, we retain only the most semantically aligned phrase prototype by taking a maximum over the phrase dimension:

(9) $$m_{j}^{k}=\max_{1\leq i\leq N_{p}}\;{\left[M^{k}\right]}_{ij}.$$

Here, $m_{j}^{k}$ captures the best phrase match of object prototype *j* at frame *k*; it suppresses weakly related phrases and prevents irrelevant phrases from lowering the matching score. Since objects often persist over time, the same semantic entity may exhibit high similarity to the same phrase across multiple frames. Accordingly, for each object prototype we aggregate its per-frame best match scores along the temporal axis and take the maximum response over the entire clip:

(10) $$m_{j}=\max_{1\leq k\leq K}\;m_{j}^{k}.$$

We scan the segment to identify, for each object prototype, its single strongest alignment response to the set of phrase prototypes. Equivalently, a high match to any phrase in any frame is sufficient to treat the object as mentioned by the query, thereby attenuating the impact of occlusion, motion blur, and transitional dynamics in the remaining frames. Finally, we average the maximal responses of all object prototypes to obtain the object-phrase matching score for the video segment:

(11) $$S_{op}=\frac{1}{N_{o}}\sum_{j=1}^{N_{o}}m_{j}=\frac{1}{N_{o}}\sum_{j=1}^{N_{o}}\max_{1\leq k\leq K}\max_{1\leq i\leq N_{p}}{\left[P_{p}\cdot {\left(P_{o}^{k}\right)}^{⊤}\right]}_{ij}.$$

$S_{op}$ indicates whether each object prototype can find, in at least one frame within the segment, a phrase prototype that matches it semantically.

### 3.4. Event-Sentence Prototype Alignment

#### 3.4.1. Temporal Prototype Generation

**Figure 5:**
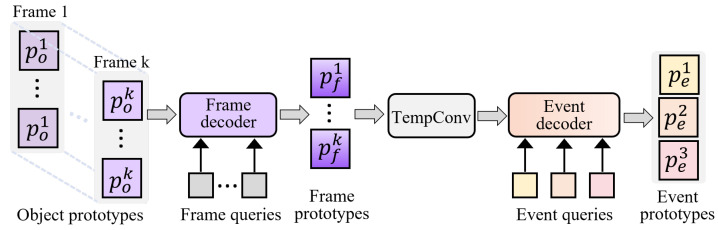
Framework of the Temporal Prototype Generation (TPG) module. Starting from object prototypes extracted at each frame, TPG first employs a frame decoder to construct frame-level prototypes. Temporal convolution is then applied to model inter-frame dependencies and capture action evolution over time. Finally, an event decoder aggregates temporally-aware features into event prototypes, which serve as semantic anchors for event-sentence prototype matching.

This section investigates how to organize the temporal evolution of object prototypes into a set of event prototypes with structural diversity while preserving local semantic resolution. The core idea is to progressively aggregate spatial object prototypes into frame-level prototypes and then perform temporal modeling on top of them to generate event prototypes with coherent semantics. The architecture of the Temporal Prototype Generator (TPG) is shown in Figure 5. We first design a frame decoder that aggregates the per-frame object prototypes $P_{o}^{k}\in R^{(K\times N_{o})\times D}$ into frame-level prototypes $p_{f}\in R^{K\times D}$. The decoder assigns each frame a learnable query $Q_{f}\in R^{K\times D}$, and uses linear projections to form the corresponding key $K_{o}$ and value $V_{o}$ for the object prototypes:

(12) $$K_{o}=P_{o}^{k}W_{o}^{K},\;V_{o}=P_{o}^{k}W_{o}^{V}.$$

where $W_{o}^{K},W_{o}^{V}\in R^{D\times D}$ are learnable parameter matrices. To ensure that each frame query attends only to the object prototypes within the same frame, we construct a mask matrix $M_{f}\in R^{K\times (K\times N_{o})}$ to restrict the attention scope, with elements defined as follows:

(13) $$M_{f}\left(i,j\right)=\left\{\begin{array}{cc} 0, & i\cdot N_{o}\leq j<\left(i+1\right)\cdot N_{o},\\ -\infty , & \mathrm{otherwise}. \end{array}\right.$$

With this mask, the frame-level prototypes are obtained through masked attention:

(14) $$p_{f}=\mathrm{Softmax}\;\left(M_{f}+Q_{f}K_{o}^{⊤}\right)V_{o}+Q_{f}.$$

Here, Softmax normalizes the weights row-wise, so that the frame query in row *i* distributes attention only among the $N_{o}$ object prototypes of the same frame, yielding a frame-level prototype vector that aggregates within-frame objects. Still, object-prototype aggregation alone can miss non-object signals, including scene context and background cues. Inspired by [55], we fuse the frame-level prototype with the global feature $x_{c}^{k}$ of the same frame by averaging to supplement scene-level semantic cues:

(15) $$p_{f}^{k}=\frac{p_{f}^{k}+x_{c}^{k}}{2}.$$

To ensure that event prototypes capture continuity across adjacent frames rather than degenerating into isolated frame representations, we impose a 1D temporal convolution with kernel size 3 on the frame-level prototypes to encode local context among neighboring frames. The frame-level prototypes after temporal convolution are expressed as:

(16) $${\tilde{p}}_{f}={\mathrm{Conv}}_{\mathrm{temp}}\;\left(P_{f}\right)\in R^{K\times D}.$$

Since the receptive field of the 1D convolution covers only the current frame and several neighboring frames, it introduces local temporal context without compromising frame-level details. We then obtain the frame-level prototypes with local temporal modeling via a residual connection:

(17) $${\hat{p}}_{f}=p_{f}+{\tilde{p}}_{f}.$$

After obtaining frame-level prototypes ${\hat{p}}_{f}$ with local temporal context, we develop an event decoder to learn temporal relations among frame-level prototypes and generate diverse event prototypes $P_{e}\in R^{N_{e}\times D}$ to represent rich action information in videos. We begin by applying linear projections to ${\hat{p}}_{f}$ to obtain the keys $K_{f}$ and values $V_{f}$:

(18) $$K_{f}={\hat{p}}_{f}W_{f}^{K},\;V_{f}={\hat{p}}_{f}W_{f}^{V},$$

where $W_{f}^{K},W_{f}^{V}\in R^{D\times D}$ are learnable projection matrices. We then compute event prototypes using scaled dot-product attention:

(19) $$P_{e}=\mathrm{Softmax}\;\left(\frac{Q_{e}K_{f}^{⊤}}{\sqrt{D}}\right)V_{f}+Q_{e}.$$

Here, $Q_{e}\in R^{N_{e}\times D}$ denotes the event query vectors, and $N_{e}$ is the predefined number of event prototypes. During training, each query vector represents a potential event type, and using multiple queries promotes diversity among event prototypes. For the text modality, we directly use the global token feature $y_{E}$ as the sentence prototype and align it with the event prototypes $P_{e}$. Similarly, the frame-level and event-level prototypes are dynamically generated from the current object prototypes and temporal features rather than stored as independent learnable representations. These prototypes are recomputed in each forward pass through attention-based aggregation parameterized by learnable query vectors, projection matrices, and temporal modeling modules such as temporal convolution and event decoding.

#### 3.4.2. Event-Sentence Matching

The same video segment can be described by different texts, some emphasizing fine-grained action details while others focus on overall semantics. Restricting alignment to phrase- and object-level representations can sharpen fine-grained correspondence, but it often overlooks sentence-level intent, causing the predicted boundaries to drift from the global semantics of the query. To address this issue, we use the global sentence feature $y_{E}$ produced by the text encoder as the sentence prototype and match it with the event prototype set ${\left\{P_{e}^{i}\right\}}_{i=1}^{N_{e}}$ to quantify event-level alignment. Because events differ in their semantic roles and only a few correspond clearly to the actions or states described in the query, a simple average over event prototypes can be dominated by irrelevant events, shifting the result away from the true target interval. To mitigate this effect, we define the event-sentence matching score using the maximum similarity:

(20) $$S_{es}=\max_{1\leq i\leq N_{e}}\langle y_{E},P_{e}^{i}\rangle .$$

Here, 〈·,·〉 denotes the inner product that measures the alignment between the sentence prototype and the *i*-th event prototype in the semantic space, and $N_{e}$ denotes the preset number of event prototypes. In contrast to weighted averaging over all event prototypes, the maximum similarity score directs the model toward the principal event that best explains the query instead of distributing evidence across multiple events. Ultimately, the event-sentence prototype matching score $S_{es}$ is regarded as the event-level alignment between the video segment and the query, guiding the model to attend to the interval that is most semantically consistent with the text along the timeline. Event-sentence matching provides temporal alignment and, together with object-phrase matching in the spatial dimension, forms a two-stage cross-modal alignment process from local semantics to global semantics.

### 3.5. Fusion of Matching Results

In the preceding alignment stage, the model derives per-clip matching scores along the spatial and temporal dimensions, and these two scores encode cross-modal alignment evidence at different semantic levels. However, the relative importance of spatial details and temporal structures varies depending on the query. For example, when a query emphasizes the actor or salient objects, spatial prototype alignment becomes more influential, whereas queries describing event ordering or action dynamics rely more on temporal prototype alignment. Therefore, a key step for accurate temporal grounding is to couple the two dimensions so that the final clip-level score can adaptively reflect the semantic focus of the query. Motivated by this observation, we design a dynamic fusion mechanism to integrate the two matching scores. Specifically, we first stack the object-phrase matching score $S_{op}$ and the event-sentence matching score $S_{es}$ to form a two-dimensional input vector, and then map it to a high-dimensional representation $H_{m}$ through a learnable fully connected layer, thereby enhancing the model’s ability to exploit complementarity across semantic levels. This process is formulated as follows:

(21) $$H_{m}=\mathrm{ReLU}\;\left(W_{m}\left[\begin{array}{c} S_{op},S_{es} \end{array}\right]+b_{m}\right).$$

The projected representation is then passed through a linear transform followed by Softmax normalization, yielding two adaptive weights α and β:

(22) $$\left[\begin{array}{c} \alpha ,\beta \end{array}\right]=\mathrm{Softmax}\;\left(W_{a}H_{m}+b_{a}\right).$$

Here, α and β quantify the relative contributions of spatial and temporal prototype matching, respectively. This dynamic weighting scheme, built on high-dimensional feature mapping, allows the model to learn the relative salience of different semantic levels during training and adjust the contributions of the two matching modules accordingly, improving robustness across diverse video-query settings. Given the learned weights, the composite score for each clip $v_{i}$ is defined as:

(23) $$S_{\mathrm{match}}\left(v_{i}\right)=\alpha S_{op}\left(v_{i}\right)+\beta S_{es}\left(v_{i}\right).$$

This dynamic fusion enables the model to adapt the spatial-versus-temporal semantic contributions to each specific video-query pairing.

### 3.6. Grounding Head

#### 3.6.1. Moment Candidates Generation

In video temporal grounding, constructing candidate moment features is a prerequisite for boundary prediction, and the quality of these representations directly determines the upper bound of candidate moment scores in the subsequent two-dimensional temporal map. Following Section 3.2, we segment the input video into *N* consecutive clips ${\left\{v_{i}\right\}}_{i=0}^{N-1}$, and candidate segments [a,b] are subsequently formed by pairing start and end indices within this clip sequence. Prior work commonly constructs candidate moment features using max pooling [7,8] or stacked convolutions [56]. However, candidate moment features generated by these paradigms are not explicitly aligned with the query semantics, which limits the accuracy of subsequent localization. In general, video clips that better match the query semantics are more likely to fall within the ground-truth moment and should therefore receive larger weights when constructing candidate segment features. Conversely, clips that correspond to background content or are semantically irrelevant should be suppressed. To this end, we use the composite matching score $S_{\mathrm{match}}\left(v_{i}\right)$ as a cross-modal semantic weight to guide candidate moment feature construction.

We first build a unified visual representation for each clip. Using average pooling, we aggregate the set of *K* sampled global token features ${\left\{x_{c}^{k}\right\}}_{k=1}^{K}$ from clip $v_{i}$ into a clip-level feature $f_{i}^{v}\in R^{D}$:

(24) $$f_{i}^{v}=\frac{1}{K}\sum_{k=1}^{K}x_{c}^{k},\;i=0,1,\ldots ,N-1.$$

We then treat ${\left\{f_{i}^{v}\right\}}_{i=0}^{N-1}$ as the primitive elements from which we build the feature map of all candidate segments. For a candidate interval [a,b], we apply Softmax normalization to the composite scores of clips within the interval to obtain cross-modal semantic weights $w_{i}$:

(25) $$w_{i}=\frac{\exp\;\left(S_{\mathrm{match}}\left(v_{i}\right)\right)}{\sum_{j=a}^{b}\exp\;\left(S_{\mathrm{match}}\left(v_{j}\right)\right)},\;i\in \left[a,b\right].$$

The weights encode how much each clip contributes to the query-relevant semantics for a fixed candidate moment. Based on these weights, we form the visual representation of a candidate moment [a,b], denoted as $f_{a,b}^{M}$, by a weighted aggregation of all clip features inside the interval:

(26) $$f_{a,b}^{M}=\sum_{i=a}^{b}w_{i}\;f_{i}^{v}.$$

Here, *a* and *b* are the start and end indices of a candidate moment, satisfying 0≤a≤b≤N−1. Relative to stacked convolutions, this construction selectively amplifies text-consistent clips and attenuates query-irrelevant noise. In contrast to max pooling, the weighted aggregation better preserves temporal continuity within the candidate moment. Given the candidate moment features, we follow the [19] formulation and arrange them into a two-dimensional temporal feature map $F^{M}\in R^{N\times N\times D}$, whose first two axes represent segment start and end locations and whose third axis is the feature dimension. Since valid candidates satisfy a≤b, we zero-pad entries that fall into the region where a>b in practice.

#### 3.6.2. Answer Prediction

After constructing candidate segment features, we predict the best-matching moment for the query sentence among all candidates. The pipeline proceeds in three stages: cross-modal fusion, contextual modeling, and score prediction. First, we fuse the two-dimensional temporal feature map $F^{M}$ with the query representation $f^{s}=y_{E}\in R^{D}$. Since the features are extracted by CLIP in a shared multimodal embedding space, we perform cross-modal fusion via a Hadamard product followed by Frobenius norm normalization, yielding the fused map F:

(27) $$F={∥\left(f^{s}\cdot 1^{⊤}\right)⊙F^{M}∥}_{F}.$$

Here, $1^{⊤}$ denotes the transpose of an all ones vector, ⊙ is the Hadamard product, and ${∥\cdot ∥}_{F}$ indicates Frobenius normalization. Next, we build a temporal adjacent network on the fused map F to produce a two-dimensional temporal map. The network contains $L_{c}$ convolutional layers, each with kernel size $K_{c}$, and is defined as:

(28) $$F^{\left(l+1\right)}=\mathrm{ReLU}\;\left(\mathrm{Conv}2D^{\left(l\right)}\;\left(F^{\left(l\right)}\right)\right),\;l=0,1,\ldots ,L_{c}-1,$$

where the network progressively incorporates richer context from adjacent segments. Finally, the output feature map is fed into a fully connected layer followed by a sigmoid function to produce a two-dimensional score map. We gather the valid entries of the score map into a set $P={\left\{p_{i}\right\}}_{i=1}^{C_{n}}$, where $C_{n}$ denotes the number of candidate moment. Each $p_{i}$ measures the consistency between a candidate moment and the query sentence, and the maximum score corresponds to the best-matched moment.

### 3.7. Training Objective

To jointly optimize cross-modal semantic alignment and temporal boundary prediction, we adopt a combination of a cross-modal alignment loss and a binary cross-entropy loss during training. The former enforces semantic consistency between video and text in a shared embedding space, while the latter guides the model to learn accurate temporal boundary prediction. In this process, the prototype representations are dynamically constructed from the current visual and textual features rather than stored as independent trainable parameters. Specifically, the object, phrase, frame-level, and event-level prototypes are recomputed in each forward pass through aggregation operations parameterized by learnable components, including aggregation networks, query vectors, projection matrices, and temporal modeling modules. All learnable components are initialized using standard random initialization, and the prototype representations gradually emerge from the aggregation of input features during early training stages. As optimization proceeds, the prototype structures are updated through end-to-end backpropagation under the supervision of the alignment and boundary prediction losses. To regularize the learning of prototype representations during training, several normalization and structural constraints are incorporated into the prototype construction process. These include layer normalization applied to input features to control feature scale, Softmax-based sparse aggregation to constrain prototype assignment distributions, masked attention to restrict cross-frame interference, and residual temporal modeling to stabilize temporal feature propagation. Together, these operations act as implicit regularization mechanisms that constrain the feature aggregation behavior and prevent unstable prototype assignments during optimization.

#### 3.7.1. Cross-Modal Alignment Loss

In the prototype alignment stage, the model computes a composite matching score $S_{\mathrm{match}}\left(v_{i}\right)$ for each video clip $v_{i}$ based on the spatial prototype alignment score $S_{op}$ and the temporal prototype alignment score $S_{es}$. This score reflects the alignment strength between the clip and the query text in the shared semantic space. We optimize $S_{\mathrm{match}}$ using a contrastive learning objective. To construct positive and negative sample sets, we compare the temporal interval of clip $v_{i}$, denoted as $\mathrm{Time}\left(v_{i}\right)=\left[i\cdot \delta ,\left(i+1\right)\cdot \delta \right]$, with the ground-truth $\mathrm{GT}=[g_{s},g_{e}]$. If $\mathrm{IoU}(\mathrm{Time}\left(v_{i}\right),\mathrm{GT})\geq \theta$, the clip is treated as a positive sample; otherwise, it is treated as a negative sample. The positive set P and negative set N are defined as:

(29) $$P=\left\{\mathrm{IoU}\left(\mathrm{Time}\left(v_{i}\right),\mathrm{GT}\right)\geq \theta \right\},\;N=\left\{\mathrm{IoU}\left(\mathrm{Time}\left(v_{i}\right),\mathrm{GT}\right)<\theta \right\}.$$

Next, we apply a Softmax operation to normalize the composite matching scores over all clips into a confidence distribution $c_{i}$:

(30) $$c_{i}=\frac{\exp\;\left(S_{\mathrm{match}}\left(v_{i}\right)/\tau \right)}{\sum_{j=0}^{N-1}\exp\;\left(S_{\mathrm{match}}\left(v_{j}\right)/\tau \right)}.$$

Here, τ is a temperature parameter that controls the smoothness of the distribution. The confidence $c_{i}$ reflects the model’s belief that clip $v_{i}$ is semantically matched to the query text. Based on this distribution, the cross-modal alignment loss is defined as:

(31) $$L_{\mathrm{Match}}=-\frac{1}{\left|P\right|}\sum_{i\in P}\log c_{i}=-\frac{1}{\left|P\right|}\sum_{i\in P}\log\frac{\exp\;\left(S_{\mathrm{match}}\left(v_{i}\right)/\tau \right)}{\sum_{j=0}^{N-1}\exp\;\left(S_{\mathrm{match}}\left(v_{j}\right)/\tau \right)}.$$

This objective strengthens the alignment structure by maximizing the normalized probability of positive segments, encouraging them to be closer to the query semantics in the cross-modal space while suppressing negatives by pushing them toward semantically distant regions.

#### 3.7.2. Binary Cross-Entropy Loss

To supervise boundary probabilities on the two-dimensional score map, we follow 2D-TAN [19] and use binary cross-entropy as the primary supervision signal. Concretely, for each candidate moment, we denote its temporal overlap with the ground truth as $o_{i}$ and linearly map it to a soft label $y_{i}$ using two predefined thresholds $t_{\min}$ and $t_{\max}$:

(32) $$y_{i}=\left\{\begin{array}{cc} 0, & o_{i}\leq t_{\min},\\ \frac{o_{i}-t_{\min}}{t_{\max}-t_{\min}}, & t_{\min}<o_{i}<t_{\max},\\ 1, & o_{i}\geq t_{\max}. \end{array}\right.$$

This label reflects the semantic proximity between a candidate moment and the ground truth. Given the predicted confidence $p_{i}$ for each candidate moment on the two-dimensional score map, the binary cross-entropy loss is defined as:

(33) $$L_{\mathrm{BCE}}=-\frac{1}{C_{n}}\sum_{i=1}^{C_{n}}y_{i}\log p_{i}+\left(1-y_{i}\right)\log\left(1-p_{i}\right).$$

By using soft labels, this loss provides coarse-to-fine temporal supervision, enabling the model to learn continuous boundary variation patterns across temporal scales and yielding smoother and more stable localization results. We adopt a weighted joint training strategy that combines the two losses into a unified optimization objective:

(34) $$L_{\mathrm{Total}}=L_{\mathrm{BCE}}+\lambda \;L_{\mathrm{Match}}.$$

Here, λ is a balancing coefficient that controls the relative contribution of the semantic alignment term to the overall objective.

## 4. Experiments

### 4.1. Datasets

To comprehensively evaluate the effectiveness and generalizability of the proposed hierarchical prototype matching framework, we conduct experiments on three widely used benchmark datasets for video temporal grounding: Charades-STA, ActivityNet Captions, and TACoS. These datasets are commonly adopted in the literature, enabling rigorous and fair comparisons with existing approaches.

Charades-STA [7] is an extended version of the Charades action recognition dataset that is specifically designed for video temporal grounding. It contains 5338 videos and 12,408 query-moment pairs for training, as well as 1334 videos with 3720 query-moment pairs for testing. The original Charades dataset was recorded using RGB cameras in indoor environments, where participants perform scripted activities. A notable characteristic of Charades-STA is its high semantic density, as each video is associated with multiple overlapping or even conflicting queries. This property often leads to ambiguous event boundaries and poses challenges for precise cross-event alignment.

ActivityNet Captions [57] is a large-scale dataset comprising 19,209 videos and over 72,000 annotated video-sentence pairs. Specifically, it is divided into 37,417 training pairs, 17,505 validation pairs, and 17,031 testing pairs. Compared with Charades-STA, video segments in ActivityNet Captions are typically longer and cover a broader range of events with more complex semantic structures; individual sentences often describe multi-event sequences and nuanced temporal relations. These characteristics make the dataset suitable for evaluating the generalization ability and temporal reasoning capability of the proposed event-level prototypes.

TACoS [58], derived from the MPII Cooking Activities dataset, consists of 127 videos and 18,818 video-sentence pairs, partitioned into 10,146 for training, 4589 for validation, and 4083 for testing. Videos in TACoS are on average over four minutes long and exhibit highly similar event semantics (e.g., “cutting tomato” versus “cutting onion”), which makes fine-grained semantic discrimination particularly challenging. This property aligns well with the object-phrase prototype matching strategy in our framework and provides a valuable testbed for evaluating its ability to capture fine-grained semantic distinctions.

### 4.2. Experimental Settings

#### 4.2.1. Evaluation Metrics

Following existing video temporal grounding works, we evaluate performance using two main metrics.

mIoU: This metric measures the mean Intersection-over-Union (IoU) between the predicted temporal intervals and the corresponding ground-truth intervals. By averaging IoU over all queries in the test set, mIoU reflects the overall localization accuracy of the model.

Recall: We adopt “Rank@n,IoU=m”, as the evaluation metrics, following [7]. The “Rank@n,IoU=m” represents the percentage of language queries having at least one result whose IoU between top−n predictions with ground-truth is larger than m. In our experiments, we reported the results of n=1 and $m\in \left\{0.3,0.5,0.7\right\}$.

#### 4.2.2. Implementation Details

Because the datasets differ substantially in event density, action duration, and scene transition pace, we do not adopt a single configuration for clip partitioning, frame overlap, or frame sampling. Instead, these settings are adapted to the characteristics of each dataset to ensure that prototypes are constructed on a stable temporal structure. Following previous work [19], we set the clip length to T=8 with no frame overlap (O=0) for Charades-STA, where events are relatively short and temporally discrete. For ActivityNet Captions, due to longer events and smoother temporal transitions, we use T=16 without frame overlap (O=0) to preserve semantic completeness within each clip. For TACoS, where event density is high and semantic continuity is strong, we set T=16 with substantial frame overlap (O=12, approximately 75% overlap) to enhance the model’s ability to capture events that span across adjacent clips.

Within each clip, we uniformly sample K=8 frames. We adopt CLIP (ViT-B/32) [54] as the multimodal backbone and initialize it with pre-trained weights. Following the spatial and temporal modeling requirements described earlier, we set the numbers of object, phrase, and event prototypes to $N_{o}=16$, $N_{p}=12$, and $N_{e}=3$, respectively. All prototypes are 512-dimensional, and these hyperparameters are kept fixed across datasets to maintain architectural consistency. We train the model using the Adam optimizer [59] with a learning rate of $1\times 10^{-4}$ and a batch size of 32 for 100 epochs, while setting the loss balancing coefficient λ to 0.5. During inference, candidate scores are post-processed using non-maximum suppression (NMS) [60] with a threshold of 0.5 to remove redundant intervals and produce the final predictions.

All experiments were conducted using a workstation equipped with an NVIDIA RTX 4090 GPU (NVIDIA Corporation, Santa Clara, CA, USA). The implementation was based on Python 3.8 and PyTorch 1.13.0 (Meta Platforms, Inc., Menlo Park, CA, USA). The video frames were extracted using FFmpeg (Version 4.3.1).

### 4.3. Comparison to State-of-the-Art Methods

#### 4.3.1. Comparative Methods

We select a set of representative VTG methods as baselines and categorize them into two groups according to their cross-modal alignment strategies: global alignment and local alignment. Global alignment methods learn a shared semantic space between whole videos and complete sentences, typically via global representation modeling and cross-modal fusion. In contrast, local alignment methods emphasize fine-grained correspondence by matching local visual regions (or frame-level cues) with words/phrases to better capture complex actions.

Global alignment methods: VSLNet [21], 2D-TAN [19], GDP [32], CPN [23], DCM [33], CI-MHA [61], MMN [20], PS-VTG [34], ViGA [35], D-TSG [36], D3G [37], and MRTNet [22].
Local alignment methods: LGI [25], MAT [38], DRN [39], LCNet [40], CBLN [24], SeqPAN [27], MGSL [28], PFU [41], VDI [42], and MESM [26].

#### 4.3.2. Quantitative Comparison

As shown by the comparative results in Table 1, Table 2 and Table 3, our method achieves strong and consistent performance across all three benchmark datasets. On ActivityNet Captions, our approach attains state-of-the-art results on the primary metrics. At IoU=0.7, it improves upon D-TSG [36] by roughly two percentage points and further outperforms MAT [38] by about four percentage points. This trend is consistent with the characteristics of ActivityNet Captions: the captions are relatively detailed and often describe objects and actions with clear phrase boundaries. Such informative queries facilitate the formation of stable semantic links from local evidence to higher-level event semantics, making prototype alignment more effective when the textual description is sufficiently specific. The results on TACoS are also informative. At IoU=0.3, our method outperforms CPN [23], a representative global-alignment approach. In terms of overall performance, it remains slightly below MESM [26], a strong local alignment method. Taken together, the results across the three benchmarks suggest that our method benefits most from informative queries with clear semantic structures. When the semantics are sparse, the performance margin narrows, while the method remains stable.

Table 1 shows that on Charades-STA our method remains competitive, but the advantage is less pronounced than on the other two benchmarks. This outcome aligns with the nature of Charades-STA, where indoor clips are visually homogeneous and temporally smooth, and the queries are brief with limited object vocabulary and weak descriptive modifiers. This sparsity in both the visual stream and the textual descriptions makes it difficult for object and phrase prototypes to extract representative semantic cues from local segments, and it also hinders event prototypes from forming robust representations when clear temporal-event boundaries are absent. Nevertheless, for candidate-segment construction, our method still yields a clear advantage over approaches such as 2D-TAN [19] and MMN [20] that rely on max-pooling to build video representations.

As shown in Table 2 and Table 3, our method outperforms global-alignment approaches such as D-TSG [36], D3G [37], CPN [23], and MRTNet [22] on multiple evaluation metrics. These methods typically rely on holistic representations over long temporal spans for cross-modal matching, which helps capture long-range semantic structures expressed in the query sentence. However, when alignment is dominated by global features, fine-grained differences among candidate segments tend to be attenuated, leaving limited representational capacity for local actions, short-term interactions, or subtle details. Consequently, global methods are more prone to misalignment when queries involve complex semantic structures, dense entities, or rapidly changing actions. In contrast, local-alignment methods such as SeqPAN [27], MAT [38], MGSL [28], and VDI [42] align sentences and videos within restricted temporal and spatial neighborhoods and can therefore capture fine details more sensitively. Yet these approaches commonly adopt a single-granularity matching strategy, for example aligning frames to words or local segments to the full sentence. When a query simultaneously refers to multiple entities and events, or when the video exhibits abrupt action changes, local features can become overly sensitive to noise and outliers, which degrades cross-modal alignment. Our method models alignment at two semantic granularities. Object-phrase prototypes constrain visual attention in the spatial dimension, focusing the model on query-relevant entities, while event-sentence prototypes further strengthen the correspondence between action trajectories and sentence semantics in the temporal dimension. This design yields more stable alignment for long and compositional queries.

**Table 1:** Comparison of our method with existing methods on the Charades-STA dataset.

Type	Method	Rank@1			mIoU
		IoU=0.3	IoU=0.5	IoU=0.7	
Global Alignment	VSLNet [21]	70.46	54.19	35.22	50.02
	2D-TAN [19]	57.31	39.70	23.31	39.23
	GDP [32]	54.54	39.47	18.49	–
	CPN [23]	64.41	46.08	25.06	43.90
	DCM [33]	–	47.80	28.00	43.10
	CI-MHA [61]	69.87	54.68	35.27	–
	MMN [20]	–	47.31	27.28	–
	PS-VTG [34]	–	39.22	20.17	–
	ViGA [35]	60.22	36.72	17.20	38.62
	D-TSG [36]	–	65.05	42.77	–
	D3G [37]	–	41.64	19.60	–
	MRTNet [22]	59.23	44.27	25.88	40.59
Local Alignment	DRN [39]	–	42.90	23.68	–
	LCNet [40]	59.60	39.19	18.87	38.94
	CBLN [24]	–	43.67	24.44	–
	SeqPAN [27]	73.84	60.86	41.34	53.92
	MGSL [28]	–	63.98	41.03	–
	PFU [41]	71.57	54.66	28.34	48.65
	VDI [42]	–	46.47	28.63	41.60
	UniVTG [62]	70.81	58.01	35.65	50.10
	MS-DETR [63]	71.34	59.62	36.48	50.59
Ours		69.91	49.32	33.68	45.03

**Table 2:** Comparison of our method with existing methods on the ActivityNet Captions dataset.

Type	Method	Rank@1			mIoU
		IoU=0.3	IoU=0.5	IoU=0.7	
Global Alignment	2D-TAN [19]	59.45	44.51	26.54	43.29
	VSLNet [21]	63.16	43.22	26.16	43.19
	GDP [32]	56.17	39.27	–	39.80
	CI-MHA [61]	61.49	43.97	25.13	–
	CPN [23]	62.81	45.10	28.10	45.70
	DCM [33]	–	44.90	27.70	43.30
	D-TSG [36]	–	54.29	33.64	–
	MMN [20]	65.05	48.59	29.26	–
	PS-VTG [34]	59.71	39.59	21.98	–
	ViGA [35]	59.61	35.79	16.96	40.12
	D3G [37]	58.25	36.68	18.54	–
	MRTNet [22]	60.71	45.59	28.07	44.54
Local Alignment	LGI [25]	58.52	41.51	23.07	41.13
	DRN [39]	58.52	41.51	23.07	43.13
	SeqPAN [27]	61.65	45.50	29.37	45.11
	CBLN [24]	66.34	48.12	27.60	–
	MAT [38]	–	48.02	31.78	–
	LCNet [40]	48.49	26.33	–	34.29
	MGSL [28]	–	51.87	31.42	–
	VDI [42]	–	32.35	16.02	34.32
	PFU [41]	59.63	36.35	16.61	40.15
Ours		67.73	56.81	35.48	46.03

**Table 3:** Comparison of our method with existing methods on the TACoS dataset.

Type	Method	Rank@1			mIoU
		IoU=0.3	IoU=0.5	IoU=0.7	
Global Alignment	VSLNet [21]	29.61	24.27	20.03	24.11
	2D-TAN [19]	37.29	25.32	13.32	25.19
	GDP [32]	24.14	–	–	16.18
	CPN [23]	47.69	36.33	–	34.49
	D-TSG [36]	46.32	35.91	–	–
	PS-VTG [34]	23.64	10.00	3.35	–
	ViGA [35]	19.62	8.85	3.22	15.47
	MMN [20]	38.57	27.24	–	–
	D3G [37]	27.27	12.67	4.70	–
	MRTNet [22]	37.81	26.01	14.95	26.29
Local Alignment	DRN [39]	–	23.17	–	–
	CBLN [24]	38.98	27.65	–	–
	SeqPAN [27]	31.72	27.19	21.65	25.86
	MAT [38]	48.79	37.57	–	–
	MGSL [28]	42.54	32.27	–	–
	MESM [26]	52.69	39.52	–	36.94
	UniVTG [62]	51.44	34.97	17.35	33.60
	MS-DETR [63]	53.16	39.65	23.42	37.01
Ours		49.65	34.77	21.86	33.89

### 4.4. Ablation Study

#### 4.4.1. Effect of Individual Components

This section presents an ablation study with several variants to quantify the contributions of the key components in our framework, including Object-Phrase Prototype Matching (OPPM) and Event-Sentence Prototype Matching (ESPM). All experiments are conducted on ActivityNet Captions, and the results are reported in Table 4. We first examine the performance when only OPPM or only ESPM is enabled. The results show that removing either module leads to a substantial performance drop. With only OPPM, the model can suppress irrelevant regions via object-phrase relations. However, it lacks explicit modeling of action evolution, making it difficult for candidate segments to remain semantically consistent with the query over longer time spans. With only ESPM, the model can aggregate event information within video segments, but without object-level local semantic support, boundary predictions become less stable in segments with dense entities or alternating actions. When both prototype modules are present, local entity cues and temporal action structure act jointly, allowing cross-modal alignment to leverage both fine-grained evidence and global semantics.

Next, we analyze how alternative prototype generation schemes affect performance. If event prototypes are derived directly from frame features (⟲TPG), they lose the entity-grounded local semantics distilled by object prototypes and become overly driven by appearance cues. If they are built only from object features (⟲SPG), inter-frame dependencies are insufficiently modeled and temporal action evolution is overlooked. Both variants degrade alignment performance, indicating that event prototypes must integrate object cues with cross-frame aggregation to remain stable. Moreover, replacing spatial prototype matching with token-to-token alignment (P–P) or object-to-word alignment (O–W) also leads to a marked performance drop. P–P fails to aggregate semantics across frames and produces fragmented event cues. O–W ignores sentence-level structure, making action-text correspondence ambiguous.

Third, we assess how the spatial and temporal decoders influence prototype construction. Removing the frame decoder (–F), the attention mask (–M), or the residual connection (–R) leads to performance degradation to varying degrees. Removing –F undermines object-level spatial coherence and increases the model’s sensitivity to local distractions during object-phrase alignment. Removing –M weakens inter-frame coupling and disrupts the temporal integration of event prototypes. Removing -R hampers feature propagation and destabilizes temporal modeling, particularly in longer videos.

Finally, we compare two alternative global alignment schemes, namely global frame-feature alignment (F–W) and mean pooling of frame features ($\bar{F}$–S). Both choices lead to substantial performance degradation. The former struggles to preserve object-level details, whereas the latter suppresses action dynamics, leaving alignment without a clear local semantic basis. We further evaluate a variant that replaces the max-based matching with mean aggregation (mean). This variant also leads to performance degradation across all evaluation metrics, likely because mean aggregation dilutes the most discriminative object–phrase correspondences by averaging similarities over many irrelevant prototypes and frames.

#### 4.4.2. Analysis of Prototype Configuration

This subsection examines how different prototype configurations affect model performance, with results reported in Table 5. The candidate ranges for the numbers of prototypes are determined according to the structural roles of the three prototype levels and the statistical characteristics of VTG datasets. For object prototypes, CLIP represents each frame using 49 spatial tokens (image patches), while only a few salient entities typically appear within a frame; therefore, we evaluate moderate spatial compression levels (6, 12, and 16). For phrase prototypes, queries in Charades-STA, ActivityNet Captions, and TACoS usually contain multiple semantic components (objects, actions, and relations), so we evaluate 12 and 18 phrase prototypes to capture these compositions. For event prototypes, queries generally refer to one or a few actions within a short temporal interval; thus, a small range (1–4) is sufficient to model diverse event structures.

The results indicate that suboptimal prototype settings can substantially degrade performance. With an insufficient number of spatial prototypes, the model fails to represent the diversity of object instances; the matching becomes sparse, and local alignment accuracy deteriorates. By contrast, an excessive number of phrase prototypes induces competition over a limited set of object prototypes, which weakens the semantic aggregation associated with each prototype. The number of event prototypes also matters: too few may under-cover distinct action segments, while too many may over-segment the temporal signal and make each event prototype less stable semantically. Among all configurations, the setting 16,12,3 performs best. Compared with the preceding groups, this configuration sharpens entity granularity through object prototypes, curbs text-side over-fragmentation by moderately reducing phrase prototypes, and keeps event prototypes at a moderate scale. As a result, alignment among entities, phrases, and event segments becomes clearer.

#### 4.4.3. Analysis of the Fusion Mechanism

To further assess how object-phrase matching and event-sentence matching interact under different query semantics, we compare static and dynamic fusion strategies on the ActivityNet Captions dataset. In static fusion, the two matching scores are linearly combined using preset weights, whereas dynamic fusion learns the weights during training and adaptively adjusts the contribution of each mechanism according to the query content and video structure. Table 6 summarizes the results of these fusion variants. When α=0, the model relies solely on event-sentence matching and achieves the weakest performance. This observation suggests that, without entity-level constraints, boundary predictions become more prone to drift. As the weight on object-phrase matching increases, the IoU=0.7 metric improves steadily, highlighting the role of entity cues in fine-grained alignment. However, excessive emphasis on spatial alignment is not optimal. When α approaches 1.0, performance at IoU=0.5 decreases slightly, indicating that relying exclusively on local entities cannot fully capture event semantics. Static fusion attains its best overall performance when α=0.5, where spatial and temporal cues contribute in a more balanced manner. Under this setting, the model can leverage both entity-level details and the event sequence, leading to strong alignment accuracy across metrics.

Dynamic fusion improves all evaluation metrics and surpasses static fusion by approximately two percentage points at IoU=0.5. This result suggests that, as query semantics vary, automatically adjusting the relative weights of the two prototype types better captures cross-level semantic dependencies. Consequently, the resulting matching score aligns more closely with the event structure and semantic intent expressed in the query sentence.

#### 4.4.4. Parameter Sensitivity

**Figure 6:**
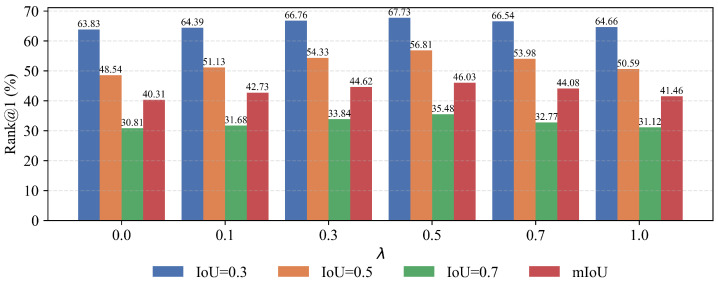
Parameter sensitivity analysis of the loss balancing coefficient λ on the ActivityNet Captions dataset. The figure reports the performance variations under different λ values, reflecting the trade-off between the cross-modal alignment loss and the boundary prediction loss.

In the joint objective, the parameter λ controls the relative weight of the cross-modal alignment loss $L_{\mathrm{Match}}$ and the binary cross-entropy loss $L_{\mathrm{BCE}}$, thus determining whether the training objective emphasizes semantic alignment or temporal boundary accuracy. To identify a reasonable balance, we evaluate λ∈{0.0,0.1,0.3,0.5,0.7,1.0} on ActivityNet Captions and report the results in Figure 6. When λ is small (e.g., 0 or 0.1), training relies almost exclusively on $L_{\mathrm{BCE}}$. In this regime, object and event prototypes do not yet impose strong discriminative constraints over candidate segments, so local semantic consistency is unstable and the model tends to select segments primarily by visual similarity. Consequently, performance remains at a relatively low level. As λ increases to 0.5, the influence of $L_{\mathrm{Match}}$ strengthens, enabling object-phrase matching to more reliably suppress segments irrelevant to query-mentioned entities and event-sentence matching to induce temporally coherent action semantics, which improves candidate ranking with respect to the query. When λ exceeds this point, $L_{\mathrm{Match}}$ dominates and $L_{\mathrm{BCE}}$ is weakened; the model focuses on semantic separation while boundary precision is under-constrained, and performance at high IoU deteriorates noticeably. Overall, setting λ around 0.5 yields a stable synergy between prototype-level semantic consistency and boundary learning, allowing both object-phrase and event-sentence matching signals to contribute effectively to candidate filtering and ranking, thereby achieving the best temporal grounding performance.

#### 4.4.5. Analysis of Kernel Size

**Figure 7:**
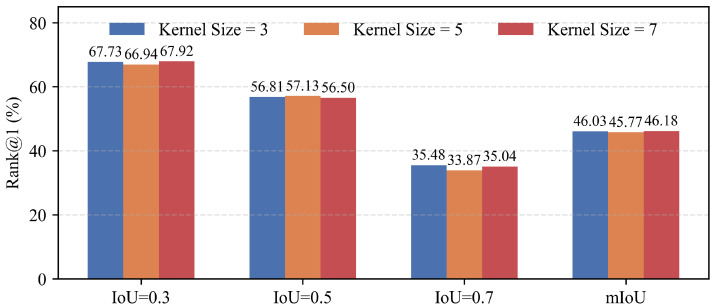
Performance comparison of temporal convolution kernel sizes (3, 5, and 7) on the ActivityNet Captions dataset.

To examine the effect of temporal receptive field design, we conduct a sensitivity analysis on the kernel size of the temporal convolution used in the Temporal Prototype Generation module. Specifically, we evaluate kernel sizes of 3, 5, and 7 on the ActivityNet Captions dataset while keeping all other settings unchanged. The results are illustrated in Figure 7.

As shown in the figure, the performance differences among the three configurations are relatively small across all evaluation metrics. Increasing the kernel size does not yield consistent improvements in temporal grounding accuracy. This behavior can be attributed to the fact that the temporal convolution in our framework operates on clip-level prototypes constructed from uniformly sampled frames, where local temporal patterns have already been captured. Larger kernels therefore provide only limited additional context while potentially introducing excessive temporal smoothing. Based on these observations, we adopt a kernel size of 3 as a balanced choice for modeling temporal evolution while maintaining sensitivity to precise temporal boundaries.

### 4.5. Efficiency Analysis

To evaluate the computational efficiency of the proposed framework, we report the total parameter count, floating-point operations (FLOPs), and inference latency under the same experimental settings used in our evaluations. The results are summarized in Table 7. All experiments were conducted on a workstation equipped with an NVIDIA RTX 4090 GPU. The model was implemented using Python 3.8 and PyTorch 1.13.0. Video frames were extracted using FFmpeg (version 4.3.1), and the inference time was averaged over the entire test split under identical hardware and batch configurations.

It is worth noting that the CLIP backbone remains frozen during both training and inference, and visual features are extracted offline. Consequently, the trainable components mainly consist of the prototype generation and matching modules, whose computational overhead is relatively small. In practice, the prototype matching network contributes less than 5 GFLOPs, indicating that the additional computational cost introduced by the hierarchical prototype alignment is lightweight compared with typical video temporal grounding architectures.

As shown in Table 7, the parameter size of the model is largely dominated by the backbone, while the learnable modules account for only a small fraction of the overall capacity. Since the model architecture remains unchanged across datasets, the parameter count is consistent, while FLOPs and inference latency vary slightly due to different clip partitioning and temporal sampling strategies.

### 4.6. Qualitative Analysis

#### 4.6.1. Visualization of Prototypes

**Figure 8:**
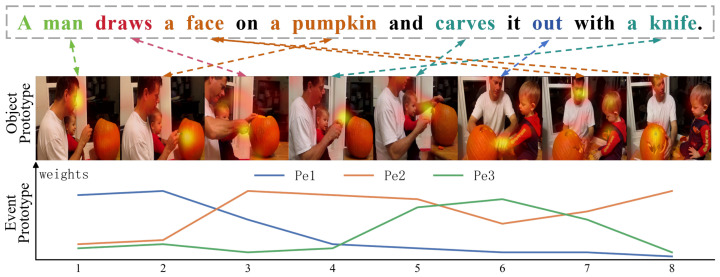
Visualization of object and event prototypes. We sample eight frames from the video, where object prototypes are illustrated as highlighted response regions aligned with the phrase. The temporal weights of event prototypes, computed by the cross-attention mechanism in Equation (19), are plotted as line curves to reflect their activation patterns over time. Best viewed in color.

In this section, we present qualitative visualizations of both spatial and event prototypes, as summarized in Figure 8. We begin by rendering object prototypes on individual frames, which show that irrelevant regions are largely suppressed and that the retained high-response areas align locally and explicitly with the corresponding textual phrases. Building on this observation, we further compute, according to Equation (19), the cross-frame attention weights for three event queries over eight video frames and visualize them using line plots. The three event prototypes exhibit clear differences along the temporal axis. For example, event prototype 1 concentrates its attention on the first two frames, capturing the action of drawing on the pumpkin; event prototype 2 attains the highest weights over frames 3–5, aligning with carving using a knife; and event prototype 3 peaks around frame 6, corresponding to the stage in which the interior is removed. These observations indicate that the model effectively learns temporal relations and that event prototypes can represent diverse semantic patterns.

#### 4.6.2. Visualization of Score Map

**Figure 9:**
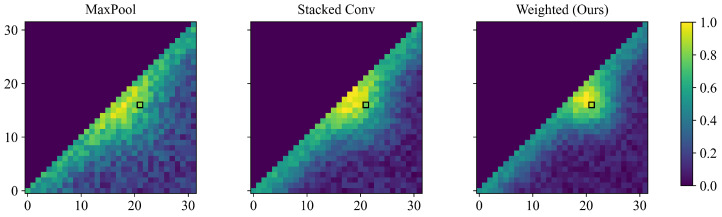
Visualization of two-dimensional score maps under different candidate moment feature constructions. From left to right, the score maps are generated using max pooling, stacked temporal convolutions, and the proposed weighted construction, respectively. Brighter regions indicate higher matching confidence between candidate moments and the query. The black box indicates the ground-truth moment. Compared with conventional designs, our method produces a more concentrated and discriminative score distribution, leading to a clearer ranking structure and more stable boundary localization. Best viewed in color.

By comparing the two-dimensional score maps produced under different constructions of candidate moment features, we can directly observe how the three methods differ in their ranking patterns, as shown in Figure 9. When candidate moment features are constructed using max pooling, local frame activations are amplified, and a single salient location can yield nearly identical scores for a large set of candidates that span it; this widens the ranking distribution, weakens inter-segment discrimination, and destabilizes boundary prediction under local perturbations. Stacked temporal convolutions partially mitigate this over-amplification effect, but the resulting moment features are still largely governed by appearance similarity; consequently, videos with visually similar actions produce overly smooth score maps whose ranking boundaries are not sharply constrained by semantics. By contrast, the proposed approach aligns candidate moment features with the query through matching results, which sharpens the score distribution by concentrating probability on text-relevant candidates while rapidly suppressing irrelevant ones. With a more decisive ranking distribution, the model becomes less susceptible to interference from neighboring candidates when selecting the best moment, and boundary prediction becomes more stable.

#### 4.6.3. Grounding Results

**Figure 10:**
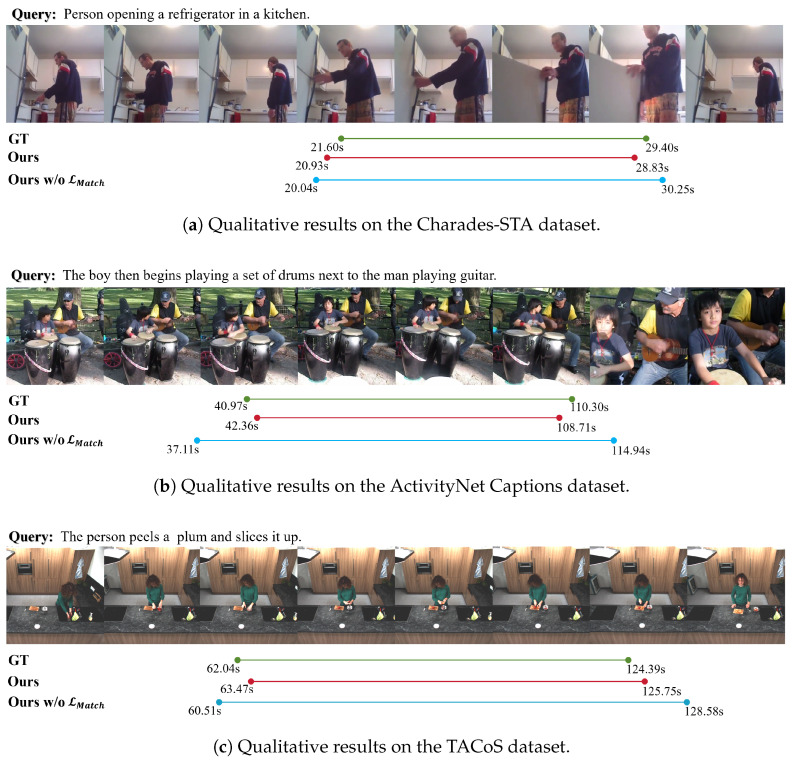
Temporal grounding visualizations on three benchmark datasets.

This section presents qualitative visualizations of grounding results on representative videos from three benchmarks, comparing the full model with a variant without the cross-modal alignment loss, as illustrated in Figure 10. Overall, these visualizations highlight the role of prototype matching in stabilizing boundary estimation under different data conditions.

In the Charades-STA example (Figure 10a), the short indoor clip contains small-scale daily actions, where object and event prototypes capture limited cues; therefore, removing the cross-modal alignment loss causes little change in prediction. In this setting, prototypes mainly help maintain stable candidate ranking.

By contrast, ActivityNet Captions videos typically involve multiple objects and concurrent events. As shown in Figure 10b, the video includes children and adults with objects such as a drum and a guitar, while drumming and instrument playing occur simultaneously. Object prototypes align with phrase-level descriptions and event prototypes with sentence-level semantics, concentrating relevant candidates while suppressing visually similar yet irrelevant intervals. Without the alignment loss, predicted boundaries drift toward nearby background segments, indicating that cross-modal alignment is important for candidate ranking in complex scenes.

TACoS videos contain long cooking procedures with clear temporal structure and ordered action steps. The case in Figure 10c shows two successive actions, peeling and cutting into pieces, which have weak visual contrast but span a long duration. The full model uses event prototypes to maintain semantic linkage over time, concentrating candidate rankings around the main action trajectory. Without the cross-modal alignment loss, the model is more easily distracted by adjacent segments and produces boundary spillover, showing that event prototypes provide useful temporal constraints in this scenario.

### 4.7. Case Study

**Figure 11:**
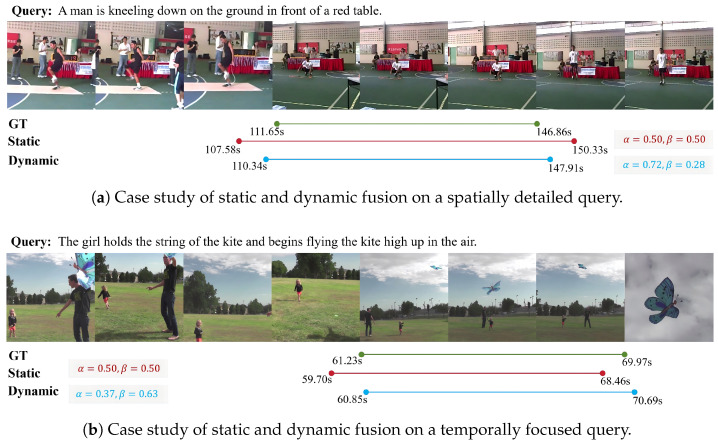
Temporal grounding visualizations on three benchmark datasets.

To further examine the behavior of the dynamic fusion mechanism under different query semantics, we present two representative examples in Figure 11. The first query mainly emphasizes spatial object-level cues (e.g., objects and spatial relations), while the second query focuses on temporal event evolution. As shown in Figure 11a, the dynamic fusion strategy assigns a higher spatial weight for the spatially detailed query, leading to a prediction closer to the ground-truth segment compared with static fusion. In contrast, for the temporally focused query in Figure 11b, the model increases the temporal matching weight, allowing event-level alignment signals to dominate the final grounding decision. These examples illustrate that the dynamic fusion mechanism can adaptively adjust the relative contributions of spatial and temporal prototype matching according to query semantics, thereby improving temporal grounding accuracy compared with fixed-weight fusion.

## 5. Conclusions

We propose a hierarchical prototype alignment framework for video temporal grounding that decomposes cross-modal alignment into complementary object-phrase and event-sentence alignments. In the object-phrase alignment stage, we generate spatial prototypes to emphasize discriminative regions and strengthen fine-grained, entity-level correspondence. In the event-sentence alignment stage, we progressively aggregate object cues over time to form event prototypes, enabling coherent alignment with sentence semantics and improving robustness under complex event compositions. Beyond performance gains, this study highlights the value of coupling local entity evidence with temporal event structure for stable grounding, and it offers a principled direction for improving VTG models through structured alignment design rather than purely heuristic architectural tuning.

## Figures and Tables

**Table 4:** Ablation results of the proposed method on the ActivityNet Captions dataset.

ID	OPPM		ESPM		Rank@1			mIoU
	SPG	Matching	TPG	Matching	IoU=0.3	IoU=0.5	IoU=0.7	
1	✗	✗	✓	✓	61.39	52.93	32.91	42.05
2	✓	✓	✗	✗	63.39	53.88	33.43	43.35
3	⟲TPG	✓	✓	✓	64.06	54.39	34.13	43.89
4	✓	✓	⟲SPG	✓	63.39	53.91	33.82	43.93
5	✓	P–P	✓	✓	62.73	53.42	32.52	42.97
6	✓	O–W	✓	✓	62.37	52.91	32.21	42.57
7	✓	✓	–F	✓	64.73	54.37	33.74	43.81
8	✓	✓	–M	✓	65.40	54.86	34.04	44.27
9	✓	✓	–R	✓	64.06	54.88	33.13	44.89
10	✓	F–W	✓	✓	64.73	54.37	34.04	44.81
11	✓	✓	✓	$\bar{F}$–S	63.39	53.39	33.87	43.43
12	✓	mean	✓	✓	66.18	54.73	32.69	44.20
13	✓	✓	✓	✓	67.73	56.81	35.48	46.03

✗ indicates removing the corresponding module;✓ indicates retaining the corresponding module;⟲ indicates replacing the corresponding module.

**Table 5:** Analysis of prototype number configurations on the ActivityNet Captions dataset.

$\left\{N_{o},N_{p},N_{e}\right\}$	Rank@1			mIoU
	IoU=0.3	IoU=0.5	IoU=0.7	
{6,14,3}	60.31	51.82	32.13	41.95
{6,18,3}	61.24	52.41	33.34	42.69
{12,14,3}	63.33	54.52	33.86	43.17
{12,18,1}	62.52	53.83	32.95	42.52
{12,18,3}	63.73	54.81	33.48	43.79
{12,18,4}	62.55	53.33	32.34	42.98
{16,12,3}	67.73	56.81	35.48	46.03

**Table 6:** Comparison of fusion strategies on the ActivityNet Captions dataset.

Fusion Strategy	α	β	Rank@1			mIoU
			IoU=0.3	IoU=0.5	IoU=0.7	
Static fusion	0.0	1.0	61.39	52.93	32.91	42.05
	0.3	0.7	64.10	53.82	33.62	43.72
	0.5	0.5	65.55	54.23	34.85	44.68
	0.7	0.3	64.23	53.95	33.68	43.92
	1.0	0.0	63.39	53.88	33.43	43.35
Dynamic fusion	–	–	67.73	56.81	35.48	46.03

**Table 7:** Computational efficiency of the proposed method on three benchmark datasets.

Dataset	FLOPS (B)	Params (M)	Times (ms)
Charades-STA	1.454	162.261	130.674
ActivityNet Captions	2.275	162.261	560.992
TACoS	2.293	162.261	570.736

## Data Availability

The data used in this study are publicly available from the corresponding benchmark datasets: Charades-STA, ActivityNet Captions, and TACoS. Processed data and implementation details supporting the findings of this work are available from the corresponding author upon reasonable request.

## References

[1] Erin Liong V., Lu J., Tan Y.P., Zhou J. Cross-Modal Deep Variational Hashing. Proceedings of the IEEE International Conference on Computer Vision (ICCV).

[2] Zhang A., Fei H., Yao Y., Ji W., Li L., Liu Z., Chua T.S., Oh A., Naumann T., Globerson A., Saenko K., Hardt M., Levine S. (2023). VPGTrans: Transfer Visual Prompt Generator across LLMs. Proceedings of the Advances in Neural Information Processing Systems.

[3] Li S., Li B., Sun B., Weng Y. (2024). Towards Visual-Prompt Temporal Answer Grounding in Instructional Video. IEEE Trans. Pattern Anal. Mach. Intell..

[4] Hu R., Singh A. UniT: Multimodal Multitask Learning with a Unified Transformer. Proceedings of the IEEE/CVF International Conference on Computer Vision (ICCV).

[5] Sun J., Li Y., Fang H.S., Lu C. Three Steps to Multimodal Trajectory Prediction: Modality Clustering, Classification and Synthesis. Proceedings of the IEEE/CVF International Conference on Computer Vision (ICCV).

[6] Su S., Zhong Z., Zhang C. Deep Joint-Semantics Reconstructing Hashing for Large-Scale Unsupervised Cross-Modal Retrieval. Proceedings of the IEEE/CVF International Conference on Computer Vision (ICCV).

[7] Gao J., Sun C., Yang Z., Nevatia R. TALL: Temporal Activity Localization via Language Query. Proceedings of the IEEE International Conference on Computer Vision (ICCV).

[8] Anne Hendricks L., Wang O., Shechtman E., Sivic J., Darrell T., Russell B. Localizing Moments in Video with Natural Language. Proceedings of the IEEE International Conference on Computer Vision (ICCV).

[9] Chu Y.W., Lin K.Y., Hsu C.C., Ku L.W. (2021). End-to-End Recurrent Cross-Modality Attention for Video Dialogue. IEEE/ACM Trans. Audio Speech Lang. Process..

[10] Ji W., Li Y., Wei M., Shang X., Xiao J., Ren T., Chua T.S. (2021). VidVRD 2021: The Third Grand Challenge on Video Relation Detection. Proceedings of the 29th ACM International Conference on Multimedia, New York, NY, USA, 20–24 October 2021.

[11] Shang X., Li Y., Xiao J., Ji W., Chua T.S. (2021). Video Visual Relation Detection via Iterative Inference. Proceedings of the 29th ACM International Conference on Multimedia, New York, NY, USA, 20–24 October 2021.

[12] Shang X., Ren T., Guo J., Zhang H., Chua T.S. (2021). Video Visual Relation Detection. Proceedings of the 25th ACM International Conference on Multimedia, New York, NY, USA, 23–27 October 2017.

[13] Li Y., Wang X., Xiao J., Ji W., Chua T.S. Invariant Grounding for Video Question Answering. Proceedings of the IEEE/CVF Conference on Computer Vision and Pattern Recognition (CVPR).

[14] Xiao J., Yao A., Liu Z., Li Y., Ji W., Chua T.S. (2022). Video as Conditional Graph Hierarchy for Multi-Granular Question Answering. Proc. Aaai Conf. Artif. Intell..

[15] Zhong Y., Ji W., Xiao J., Li Y., Deng W., Chua T.S., Goldberg Y., Kozareva Z., Zhang Y. (2022). Video Question Answering: Datasets, Algorithms and Challenges. Proceedings of the 2022 Conference on Empirical Methods in Natural Language Processing.

[16] Liang V.W., Zhang Y., Kwon Y., Yeung S., Zou J.Y., Koyejo S., Mohamed S., Agarwal A., Belgrave D., Cho K., Oh A. (2022). Mind the Gap: Understanding the Modality Gap in Multi-modal Contrastive Representation Learning. Proceedings of the Advances in Neural Information Processing Systems.

[17] Sun C., Myers A., Vondrick C., Murphy K., Schmid C. VideoBERT: A Joint Model for Video and Language Representation Learning. Proceedings of the IEEE/CVF International Conference on Computer Vision (ICCV).

[18] Changpinyo S., Pont-Tuset J., Ferrari V., Soricut R. Telling the What While Pointing to the Where: Multimodal Queries for Image Retrieval. Proceedings of the IEEE/CVF International Conference on Computer Vision (ICCV).

[19] Zhang S., Peng H., Fu J., Luo J. (2020). Learning 2D Temporal Adjacent Networks for Moment Localization with Natural Language. arXiv.

[20] Wang Z., Wang L., Wu T., Li T., Wu G. (2021). Negative Sample Matters: A Renaissance of Metric Learning for Temporal Grounding. arXiv.

[21] Zhang H., Sun A., Jing W., Zhou J.T. (2021). Span-based Localizing Network for Natural Language Video Localization. arXiv.

[22] Ji W., Qin Y., Chen L., Wei Y., Wu Y., Zimmermann R. Mrtnet: Multi-Resolution Temporal Network for Video Sentence Grounding. Proceedings of the ICASSP 2024—2024 IEEE International Conference on Acoustics, Speech and Signal Processing (ICASSP).

[23] Zhao Y., Zhao Z., Zhang Z., Lin Z. Cascaded Prediction Network via Segment Tree for Temporal Video Grounding. Proceedings of the 2021 IEEE/CVF Conference on Computer Vision and Pattern Recognition (CVPR).

[24] Liu D., Qu X., Dong J., Zhou P., Cheng Y., Wei W., Xu Z., Xie Y. Context-aware Biaffine Localizing Network for Temporal Sentence Grounding. Proceedings of the 2021 IEEE/CVF Conference on Computer Vision and Pattern Recognition (CVPR).

[25] Mun J., Cho M., Han B. Local-Global Video-Text Interactions for Temporal Grounding. Proceedings of the 2020 IEEE/CVF Conference on Computer Vision and Pattern Recognition (CVPR).

[26] Liu Z., Li J., Xie H., Li P., Ge J., Liu S.A., Jin G. (2024). Towards balanced alignment: Modal-enhanced semantic modeling for video moment retrieval. Proceedings of the Thirty-Eighth AAAI Conference on Artificial Intelligence and Thirty-Sixth Conference on Innovative Applications of Artificial Intelligence and Fourteenth Symposium on Educational Advances in Artificial Intelligence.

[27] Zhang H., Sun A., Jing W., Zhen L., Zhou J.T., Goh S.M.R. (2021). Parallel Attention Network with Sequence Matching for Video Grounding. Proceedings of the Findings of the Association for Computational Linguistics: ACL-IJCNLP 2021.

[28] Liu D., Qu X., Di X., Cheng Y., Xu Z., Zhou P. (2022). Memory-Guided Semantic Learning Network for Temporal Sentence Grounding. Proc. Aaai Conf. Artif. Intell..

[29] Lin C., Wu A., Liang J., Zhang J., Ge W., Zheng W.S., Shen C., Koyejo S., Mohamed S., Agarwal A., Belgrave D., Cho K., Oh A. (2022). Text-Adaptive Multiple Visual Prototype Matching for Video-Text Retrieval. Proceedings of the Advances in Neural Information Processing Systems.

[30] Choi J., Gao C., Messou J.C.E., Huang J.B., Wallach H., Larochelle H., Beygelzimer A., d’Alché-Buc F., Fox E., Garnett R. (2019). Why Can’t I Dance in the Mall? Learning to Mitigate Scene Bias in Action Recognition. Proceedings of the Advances in Neural Information Processing Systems.

[31] Wang J., Ge Y., Cai G., Yan R., Lin X., Shan Y., Qie X., Shou M.Z. Object-Aware Video-Language Pre-Training for Retrieval. Proceedings of the IEEE/CVF Conference on Computer Vision and Pattern Recognition (CVPR).

[32] Chen L., Lu C., Tang S., Xiao J., Zhang D., Tan C., Li X. (2020). Rethinking the Bottom-Up Framework for Query-Based Video Localization. Proc. Aaai Conf. Artif. Intell..

[33] Yang X., Feng F., Ji W., Wang M., Chua T.S. (2021). Deconfounded Video Moment Retrieval with Causal Intervention. Proceedings of the 44th International ACM SIGIR Conference on Research and Development in Information Retrieval, New York, NY, USA, 11–15 July 2021.

[34] Xu Z., Wei K., Yang X., Deng C. (2023). Point-Supervised Video Temporal Grounding. IEEE Trans. Multimed..

[35] Cui R., Qian T., Peng P., Daskalaki E., Chen J., Guo X., Sun H., Jiang Y.G. (2022). Video Moment Retrieval from Text Queries via Single Frame Annotation. Proceedings of the 45th International ACM SIGIR Conference on Research and Development in Information Retrieval, New York, NY, USA, 11–15 July 2022.

[36] Liu D., Qu X., Hu W. (2022). Reducing the Vision and Language Bias for Temporal Sentence Grounding. Proceedings of the 30th ACM International Conference on Multimedia, New York, NY, USA, 10–14 October 2022.

[37] Li H., Shu X., He S., Qiao R., Wen W., Guo T., Gan B., Sun X. D3G: Exploring Gaussian Prior for Temporal Sentence Grounding with Glance Annotation. Proceedings of the 2023 IEEE/CVF International Conference on Computer Vision (ICCV).

[38] Zhang M., Yang Y., Chen X., Ji Y., Xu X., Li J., Shen H.T. Multi-Stage Aggregated Transformer Network for Temporal Language Localization in Videos. Proceedings of the IEEE/CVF Conference on Computer Vision and Pattern Recognition (CVPR).

[39] Zeng R., Xu H., Huang W., Chen P., Tan M., Gan C. Dense Regression Network for Video Grounding. Proceedings of the 2020 IEEE/CVF Conference on Computer Vision and Pattern Recognition (CVPR).

[40] Yang W., Zhang T., Zhang Y., Wu F. (2021). Local Correspondence Network for Weakly Supervised Temporal Sentence Grounding. IEEE Trans. Image Process..

[41] Ju C., Wang H., Liu J., Ma C., Zhang Y., Zhao P., Chang J., Tian Q. (2023). Constraint and Union for Partially-Supervised Temporal Sentence Grounding. arXiv.

[42] Luo D., Huang J., Gong S., Jin H., Liu Y. Towards Generalisable Video Moment Retrieval: Visual-Dynamic Injection to Image-Text Pre-Training. Proceedings of the 2023 IEEE/CVF Conference on Computer Vision and Pattern Recognition (CVPR).

[43] Liu C., Zhao R., Chen J., Qi Z., Zou Z., Shi Z. (2023). A Decoupling Paradigm with Prompt Learning for Remote Sensing Image Change Captioning. IEEE Trans. Geosci. Remote Sens..

[44] Liu C., Chen K., Zhang H., Qi Z., Zou Z., Shi Z. (2024). Change-Agent: Toward Interactive Comprehensive Remote Sensing Change Interpretation and Analysis. IEEE Trans. Geosci. Remote Sens..

[45] Reed S.K. (1972). Pattern recognition and categorization. Cogn. Psychol..

[46] Rosch E., Mervis C.B., Gray W.D., Johnson D.M., Boyes-Braem P. (1976). Basic objects in natural categories. Cogn. Psychol..

[47] Graf A.B.A., Bousquet O., Rätsch G., Schölkopf B. (2009). Prototype Classification: Insights from Machine Learning. Neural Comput..

[48] Snell J., Swersky K., Zemel R., Guyon I., Luxburg U.V., Bengio S., Wallach H., Fergus R., Vishwanathan S., Garnett R. (2017). Prototypical Networks for Few-shot Learning. Proceedings of the Advances in Neural Information Processing Systems.

[49] Pahde F., Puscas M., Klein T., Nabi M. Multimodal Prototypical Networks for Few-Shot Learning. Proceedings of the IEEE/CVF Winter Conference on Applications of Computer Vision (WACV).

[50] Gao T., Han X., Liu Z., Sun M. (2019). Hybrid Attention-Based Prototypical Networks for Noisy Few-Shot Relation Classification. Proc. Aaai Conf. Artif. Intell..

[51] Zhao K., Jin X., Bai L., Guo J., Cheng X., Goldberg Y., Kozareva Z., Zhang Y. (2022). Knowledge-Enhanced Self-Supervised Prototypical Network for Few-Shot Event Detection. Proceedings of the Findings of the Association for Computational Linguistics: EMNLP 2022.

[52] Hu Z., Li Z., Xu D., Bai L., Jin C., Jin X., Guo J., Cheng X. (2023). ProtoEM: A Prototype-Enhanced Matching Framework for Event Relation Extraction. arXiv.

[53] Huang Y., Yang L., Sato Y., Avidan S., Brostow G., Cissé M., Farinella G.M., Hassner T. (2022). Compound Prototype Matching for Few-Shot Action Recognition. Proceedings of the Computer Vision—ECCV 2022.

[54] Radford A., Kim J.W., Hallacy C., Ramesh A., Goh G., Agarwal S., Sastry G., Askell A., Mishkin P., Clark J., Meila M., Zhang T. (2021). Learning Transferable Visual Models From Natural Language Supervision. Proceedings of the 38th International Conference on Machine Learning, Virtual, 18–24 July 2021.

[55] Li P., Xie C.W., Zhao L., Xie H., Ge J., Zheng Y., Zhao D., Zhang Y. Progressive Spatio-Temporal Prototype Matching for Text-Video Retrieval. Proceedings of the IEEE/CVF International Conference on Computer Vision (ICCV).

[56] Zhang D., Dai X., Wang X., Wang Y.F., Davis L.S. MAN: Moment Alignment Network for Natural Language Moment Retrieval via Iterative Graph Adjustment. Proceedings of the IEEE/CVF Conference on Computer Vision and Pattern Recognition (CVPR).

[57] Krishna R., Hata K., Ren F., Fei-Fei L., Niebles J.C. Dense-Captioning Events in Videos. Proceedings of the 2017 IEEE International Conference on Computer Vision (ICCV).

[58] Rohrbach M., Regneri M., Andriluka M., Amin S., Pinkal M., Schiele B. (2012). Script data for attribute-based recognition of composite activities. Proceedings of the 12th European Conference on Computer Vision—Volume Part I, Berlin, Heidelberg, 2012.

[59] Kingma D.K., Ba J. (2024). A method for stochastic optimization. arXiv.

[60] Hosang J., Benenson R., Schiele B. Learning Non-Maximum Suppression. Proceedings of the IEEE Conference on Computer Vision and Pattern Recognition (CVPR).

[61] Yu X., Malmir M., He X., Chen J., Wang T., Wu Y., Liu Y., Liu Y. (2021). Cross Interaction Network for Natural Language Guided Video Moment Retrieval. Proceedings of the 44th International ACM SIGIR Conference on Research and Development in Information Retrieval, New York, NY, USA, 11–15 July 2021.

[62] Lin K.Q., Zhang P., Chen J., Pramanick S., Gao D., Wang A.J., Yan R., Shou M.Z. UniVTG: Towards Unified Video-Language Temporal Grounding. Proceedings of the IEEE/CVF International Conference on Computer Vision (ICCV).

[63] Ma H., Wang G., Yu F., Jia Q., Ding S. (2025). MS-DETR: Towards Effective Video Moment Retrieval and Highlight Detection by Joint Motion-Semantic Learning. Proceedings of the 33rd ACM International Conference on Multimedia, New York, NY, USA, 27–31 October 2025.

